# Polymorphisms of two histamine-metabolizing enzymes genes and childhood allergic asthma: a case control study

**DOI:** 10.1186/1476-7961-8-14

**Published:** 2010-11-01

**Authors:** Aleksandra Szczepankiewicz, Anna Bręborowicz, Paulina Sobkowiak, Anna Popiel

**Affiliations:** 1 Laboratory of Molecular and Cell Biology, Department of Pediatric Pulmonology, Allergy and Clinical Immunology, Poznan University of Medical Sciences, Poland; 2 Department of Pediatric Pulmonology, Allergy and Clinical Immunology, Poznan University of Medical Sciences, Poland

## Abstract

**Background:**

Histamine-metabolizing enzymes (N-methyltransferase and amiloride binding protein 1) are responsible for histamine degradation, a biogenic amine involved in allergic inflammation. Genetic variants of *HNMT *and *ABP1 *genes were found to be associated with altered enzyme activity. We hypothesized that alleles leading to decreased enzyme activity and, therefore, decreased inactivation of histamine may be responsible for altered susceptibility to asthma.

**Methods:**

The aim of this study was to analyze polymorphisms within the *HNMT *and *ABP1 *genes in the group of 149 asthmatic children and in the group of 156 healthy children. The genetic analysis involved four polymorphisms of the *HNMT *gene: rs2071048 (-1637T/C), rs11569723 (-411C/T), rs1801105 (Thr105Ile = 314C/T) and rs1050891 (1097A/T) and rs1049793 (His645Asp) polymorphism for *ABP1 *gene. Genotyping was performed with use of PCR-RFLP. Statistical analysis was performed using Statistica software; linkage disequilibrium analysis was done with use of Haploview software.

**Results:**

We found an association of TT genotype and T allele of Thr105Ile polymorphism of *HNMT *gene with asthma. For other polymorphisms for *HNMT *and *ABP1 *genes, we have not observed relationship with asthma although the statistical power for some SNPs might not have been sufficient to detect an association. In linkage disequilibrium analysis, moderate linkage was found between -1637C/T and -411C/T polymorphisms of *HNMT *gene. However, no significant differences in haplotype frequencies were found between the group of the patients and the control group.

**Conclusions:**

Our results indicate modifying influence of histamine N-methyltransferase functional polymorphism on the risk of asthma. The other HNMT polymorphisms and ABP1 functional polymorphism seem unlikely to affect the risk of asthma.

## Background

Histamine is a preformed mediator released during mast cell degranulation that plays a key role in the development of allergic inflammation and, subsequently, leads to atopic diseases such as bronchial asthma. Released histamine is metabolized by two enzymes: N-methyltransferase (HNMT) and diamine oxidase (amiloride binding protein 1, ABP1).

N-methylation catalyzed by cytosolic HNMT enzyme is the primary pathway for histamine bio-transformation in bronchial epithelium [1]. *HNMT *gene is located on the chromosome 2q22.1 and within the gene region, several polymorphisms have been identified. A common C314T polymorphism leading to Thr105Ile substitution was discovered by Preuss et al. [2] and it was found that less common T allele (encoding Ile) was associated with decreased HNMT enzyme activity [2,3]. Other functional SNP T939C (rs1050891) is located in the 3' untranslated region of the gene and correlates with HNMT activity, as Kim et al. [4] showed that the C allele correlated with increased stability of transcripts containing the HNMT 3' untranslated region and consequently increased enzyme activity. Both polymorphisms are in strong linkage disequilibrium. Other SNPs from the 5’-flanking region (-1637T/C, -463T/C, -411C/T) as well as 3’UTR (939A/G and 1097A/T) of *HNMT *gene have been also identified [5], however their functionality has not been elucidated yet.

ABP1 enzyme is mainly expressed in kidney, colon, placenta, thymus and seminal vesicles and plays role in the inactivation of extracellular histamine [6-8]. The *ABP1 *gene has been localized on chromosome the 7q34-36 and within the gene region several polymorphisms have been identified. Among these, His645Asp substitution (rs1049793) was found to be functional and was associated with significant decrease in the serum enzyme activity *in vivo *[9]. Other non-synonymous SNPs that were suspected to influence enzyme activity or kinetics based on UniProt database include Thr16Met (rs10156191) and Ser332Phe (rs1049742). Although they were found to slightly alter enzyme kinetics by increasing the Km of the ABP1 enzyme, no significant changes were observed in relation to the genotypes of those two SNPs [9].

The importance of genetic variation of genes related to histamine (including histamine-metabolizing enzymes *HNMT *and *ABP1*) was widely discussed in recent review on histamine pharmacogenomics where authors summarized association studies of those genes and their involvement in diverse diseases, including allergic diseases and asthma [10].

We hypothesized that polymorphisms within the HNMT and ABP1 genes responsible for individual variation of histamine metabolism might contribute to the pathophysiology of asthma. The aim of our study was to analyze a relationship between the polymorphisms of two genes encoding histamine metabolizing enzymes (*HNMT *and *ABP1*) with the predisposition to asthma in the Polish population of pediatric patients.

## Methods

### Patients’ group

The study was performed on Polish sample of 149 asthmatic patients of Caucasian origin in age from 6 to 18 years old (86 boys with a mean age of 11.8 years, SD = 3.1; 63 girls with a mean age of 12.0 years, SD = 3.8). Patients were recruited from inpatients from Wielkopolska region, considered as ethnically homogenous [11], and were treated for asthma in the Department of Pediatric Pulmonology, Allergy and Clinical Immunology of Poznan University of Medical Sciences. Asthma diagnosis was made according to GINA recommendation, based on clinical asthma symptoms and lung function test (bronchodilator responsiveness, exercise induced hyperresponsiveness); bronchodilator response was assessed 20 minutes after administration of 200 mcg of Salbutamol MDI via a holding chamber (Volumatic) and a ≥ 12% increase in FEV1 was diagnostic; bronchial hyperresponsiveness was assessed by exercise test using 6 min. run on the treadmill and a post exercise fall in FEV1 of ≥ 15% was considered positive.

Clinical diagnosis of atopy depended on current or past symptoms of atopic dermatitis, allergic rhinoconjunctivitis (seasonal or perennial) or food allergy. Atopy was confirmed when children fulfilled one of the following criteria: total IgE level higher than the upper normal limits for age; positive skin prick test to at least one aero-allergen (Dermatophagoides pteronyssinus, Dermatophagoides farinae, cat, dog, feathers, Alternaria alternata, Cladosporium herbarum; pollen: grass mix, rye, birch pollen, alder, hazel – Allergopharma, Germany). Any reaction with mean wheal diameter at least 3 mm greater than negative control was regarded positive and defined atopy. Total serum IgE level was measured by a fluoroimmunossay with Pharmacia UniCap 100 System^® ^(Pharmacia, Uppsala, Sweden) following manufacturer’s instruction. The upper limits of normal range for total IgE was age-dependent (70 kU/l for 6 yr children; 79 KU/L for 7 yr children, 89 KU/L for 8 yr children, 98 KU/L for 9 yr children, 107.0 KU/L for children of 10 years and older).

### Control group

Control group consisted of 156 healthy subjects of Caucasian origin (76 boys with a mean age of 10.8 years, SD = 2.7; 80 girls with a mean age of 10.3 years, SD = 2.9). Control subjects were also recruited from the same geographic region (Wielkopolska) from the group of carefully chosen volunteers without asthma and allergy symptoms. Any allergic diseases or asthma were excluded based on clinical examination, history, spirometry and exhaled NO measurement.

All participants as well as their parents have given written informed consent. Local ethics committee accepted the project. Study was performed in compliance with the Code of Ethics of the World Medical Association (Declaration of Helsinki).

### Genotyping

The DNA was extracted from 10 ml of EDTA anticoagulated whole blood using the salting out method [12]. The *HNMT *and *ABP1 *polymorphisms were analyzed by PCR-RFLP method. The conditions of PCR-RFLP and sequences of the primers for the *HNMT *polymorphisms (-1637C/T, -411C/T, 314C/T and 1097A/T) were used as described previously [13]. For *ABP1 *His645Asp polymorphism, the genotyping was performed according to conditions described by Garcia-Martin et al. [14].

The uncut PCR products for *HNMT *polymorphisms were digested twice to confirm the results. The quality control of RFLP analysis was also performed (15% of randomly chosen samples from both groups) and the concordance between two assays was 100%. DNA samples were randomly plated during genotyping and reactions were performed without knowing the clinical outcome of the patient.

### Statistical analysis

The Pearson’s chi-square (χ^2^) test and Fisher’s exact test were used to test differences in the genotypic and allelic (respectively) distribution in case control. The alpha level < 0.05 was considered significant. Calculations were performed using the STATISTICA version 8.0 software. The association between *HNMT *314 C/T and *ABP1 *polymorphisms and the risk of developing asthma was estimated by an odds ratio (OR) with a 95% confidence interval (CI) using demo of GraphPad InStat 3 programme. Concordance with Hardy-Weinberg law was performed using "Utility Programs For Analysis Of Genetic Linkage” application (Copyright ^© ^1988 J. Ott). We also performed linkage disequilibrium analysis of the analyzed polymorphisms of *HNMT *gene using free online software Haploview version 4.1 from the website: http://www.broadinstitute.org/haploview[15]. Power calculations were done in Quanto v.1.2.3 with OR values between 1.1 and 2.5 for two-sided associations were for *HNMT *polymorphisms: 10% for 1637, 82% for 314, 9.7% for 411, 23.8% for 1097 and 23.9% for *ABP1 *polymorphism.

## Results

Genotype distributions for all studied polymorphisms in the *HNMT *and *ABP1 *genes were in concordance with Hardy-Weinberg law in both cases and control subjects, except -1637C/T *HNMT *polymorphism in the control group (p = 0.026) and *ABP1 *His645Asp polymorphism in the group of asthmatic patients (p = 0.046).

In the analysis of genotype distribution we observed significant differences for 314C/T polymorphism, with TT genotype significantly more frequent in the group of asthmatic children in comparison to the control group (Table 1). When we analyzed our group according to gender, we also observed the association of TT genotype in the group of asthmatic boys compared to the healthy boys (p = 0.003). For the other three polymorphisms (-1637C/T, -411C/T and 1097A/T) we did not observe significant differences in genotype distribution between the group of asthmatic patients and the control group (Table 1).

**Table 1:** Genotype distributions and allele frequencies of four *HNMT *polymorphisms and one ABP1 polymorphism for asthmatic patients versus control group (figures in parentheses indicate percentages)

Gene	Polymorphism			Asthma	Control	P value
	1637			N = 139	N = 137	0.352
					
		genotypes	TT	38 (27.3)	41 (29.9)	
			CT	75 (53.9)	79 (57.7)	
			CC	26 (18.8)	17 (12.4)	
		
		alelles	T	151 (54.3)	161 (58.7)	0.303
			C	127 (45.7)	113 (41.3)	
	
	314			N = 149	N = 147	0.042*
					
		genotypes	CC	99 (66.4)	119 (80.9)	
			CT	43 (28.9)	26 (17.7)	
			TT	7 (4.7)	2 (1.4)	
		
		alelles	C	241 (80.9)	264 (89.8)	0.048*
			T	45 (19.1)	30 (10.2)	
	
HNMT	411			N = 146	N = 156	0.720
					
		genotypes	TT	7 (4.8)	5 (3.2)	
			CT	43 (29.4)	50 (32.1)	
			CC	96 (65.8)	101 (64.7)	
		
		alelles	T	57 (19.5)	60 (19.2)	1.000
			C	235 (80.5)	252 (80.8)	
	
	1097			N = 146	N = 156	0.977
					
		genotypes	CC	90 (61.6)	95 (60.9)	
			CG	51 (34.9)	55 (35.3)	
			GG	5 (3.4)	6 (3.8)	
		
		alelles	C	231 (79.1)	245 (78.5)	0.920
			G	61 (20.9)	67 (21.5)	
	
ABP1	His645Asp			N = 146	N = 156	0.530
					
		genotypes	TT	75 (51.4)	79 (54.1)	
			AT	52 (35.6)	54 (37.0)	
			AA	19 (13.0)	13 (8.9)	
		
		alelles	T	202 (69.2)	212 (72.6)	0.412
			A	90 (30.8)	80 (27.4)	

* indicates significance: χ^2 ^= 6.302, df = 2

Comparison of allele frequencies revealed that T allele of 314C/T polymorphism was significantly more frequent in the group of asthmatic patients as compared to the control group (p = 0.048). This allele was also statistically more frequent in the group of asthmatic boys (p = 0.009), but not in the group of asthmatic girls in comparison to the control subjects, male and female, respectively. The OR (95%CI) for carriers of T allele (Ile) was 1.88 (1.09-3.25) for patients with asthma. For the other three polymorphisms of *HNMT *gene we have not found any significant differences between cases and controls. The positive result for 314C/T polymorphism was confirmed further by genotyping additional group of our group of asthmatic (n = 174 altogether) and control children (n = 211 altogether) of the same Caucasian origin and the association was confirmed (p = 0.015 for genotypes; p = 0.011 for alleles, OR = 1.745, 95%CI:1.151-2.646).

For the *ABP1 *gene His645Asp polymorphism, no significant differences were in genotype distribution (p = 0.530) or allele frequencies (p = 0.412) between the asthmatic patients and the control group. The OR (95%CI) for carriers of *ABP1 *G variant (Asp) was 1.16 (0.70-1.76) in patients with asthma. No gender-specific differences were observed in our sample for the studied *ABP1 *polymorphism. Data were shown in table 1.

We have also performed analysis of the *HNMT *and *ABP1 *SNPs in regard to asthma-related phenotypes such as asthma severity, total IgE level, FEV1 and exNO measurement, however, no significant associations with any of the studied polymorphisms were observed (data not shown).

In linkage disequilibrium analysis for *HNMT *polymorphisms, we found suggestive evidence for linkage between -1637C/T and -411C/T polymorphisms (D’ = 0.95; 95%CI = 0.86-0.99; LOD = 22.5; r^2 ^= 0.295). However, comparing haplotype frequencies in this block, no significant differences were observed between the asthmatic patients and the control subjects (see table 2). Based on the four gamete rule (Wang et al.2002) that assumes linkage if the 4^th ^gamete is observed at frequency > 0.01, we found such a linkage between -411C/T and 314C/T polymorphisms (D’ = 1.0; LOD = 2.01; r^2 ^= 0.0.035). When we compared haplotype frequencies within this block, we observed that CT haplotype (C allele of -411 polymorphism and T allele of 314 polymorphism) was significantly more frequent in the group of asthmatic patients in comparison to the control subjects (p = 0.0046). However, this linkage seems unlikely, considering the distance between those two markers (37 kb) and observed association of CT haplotype with asthma probably depends only on the association of 314C/T polymorphism in our group.

**Table 2:** Comparison of haplotype frequencies for -1637 and -411 polymorphisms between asthmatics and control group

Haplotype	Frequency	Case: control ratios	χ^2^	p
TC	0.564	0.540 : 0.586	1.309	0.2525

CC	0.242	0.265 : 0.222	1.516	0.2183

CT	0.189	0.187 : 0.190	0.0090	0.9236

As both *HNMT *and *ABP1 *enzymes are involved in histamine metabolism, we analyzed both functional variants together (Thr105Ile of *HNMT *gene and His645Asp of *ABP1 *gene) to check if the two allelic variants responsible for reduced activity of both enzymes are more frequently observed in asthmatic patients. We have not found any statistically significant differences in frequencies of *HNMT *and *ABP1 *allelic variants combinations between the asthmatic patients and the control subjects (data not presented).

## Discussion

The main finding of this study is an association of 314C/T polymorphism of *HNMT *gene with asthma in the Polish population of pediatric patients which may confirm that impaired histamine metabolism caused by reduced activity of HNMT is involved in asthma pathogenesis.

This common polymorphism responsible for Thr105Ile substitution was discovered by Preuss et al. [2] and it was found that less common T allele (encoding Ile) was associated with decreased HNMT enzyme activity [2,3]. In our study, we found this T allele associated with asthma, which is consistent with the findings by Yan et al. [16], but in contrast to the results obtained by the others [17-19]. In addition, this SNP was also associated with higher risk of developing atopic dermatitis [20]. This most extensively studied polymorphism of *HNMT *gene was also involved in other disorders associated with altered histamine metabolism such as inflammatory bowel disease, neuronal degeneration and alcoholism as summarized by Garcia-Martin et al. [10]. Many inconsistent findings regarding this SNP throughout the different populations analyzed, were, in part, due to insufficient power of those studies (including the present one) to detect an association. Therefore, we aimed to combine the frequencies for particular genotypes and alleles from different populations to see if increasing sample size could be more sensitive in finding association by increasing statistical power. We have taken together the data from the genotype and allele frequencies from the other papers with both positive ([16], present study) and negative results [17-19,21] for asthmatics and control group (regardless the ethnicity) and we found that TT genotype and T allele were significantly more frequent among asthmatic patients (Table 3). Taking into account the relatively low MAF of this SNP, we have demonstrated that increasing the sample size may produce more reliable and consistent results as the power calculation for the combined sample was sufficient (78.3%) to detect an association.

**Table 3:** Genotype distributions and allele frequencies for 314C/T polymorphism of *HNMT *gene from five association studies in asthma (asthmatic patients vs. control group)

	Asthma (n = 1049)	Control group (n = 1242)	P value
CC	806 (76.8)	1011 (81.4)	0.007*
CT	221 (21.1)	219 (17.6)	
TT	22 (2.1)	12 (1.0)	

C	1833 (87.4)	2241 (90.2)	0.002*
T	265 (12.6)	243 (9.8)	

* Indicates significance: for genotypes: χ^2 ^= 9.839, df = 2; for alleles: χ^2 ^= 9.075, df = 1, OR = 1.33, 95%CI: 1.108-1.604.

The other SNPs from the 5’-flanking region (-1637T/C, -463T/C, -411C/T) as well as 3’UTR (939A/G and 1097A/T) of *HNMT *gene have been also reported [13], however their functionality has not been well described yet. The region containing -411C/T polymorphism is located in a positive regulatory sequence between nucleotides -493 and -395 [22]. For the other SNPs, no data about their possible functionality are available to our knowledge.

Among several *ABP1 *polymorphisms, one contributing to His645Asp substitution (rs1049793) was found to be functional and was associated with significant decrease in the serum enzyme activity *in vivo *[9]. In our group we did not observe an association of this polymorphism with asthma, which is consistent with the findings by Garcia-Martin et al. [19]. It is also noteworthy, that enzyme activity may be influenced by other ABP1 polymorphisms (eg. Thr16Met) and factors such as gender, with enzyme activity being significantly higher in healthy women in comparison to men, therefore ABP1 activity as a biological marker should be treated with cautious [23]. However, involvement of His645Asp SNP in histamine metabolism cannot be ignored and may be related to precise clinical phenotype rather than asthma per se.

The main limitation of the present study was the small sample size of the analyzed group. Our group (n = 149 asthmatic patients) is comparable to the group reported by Garcia-Martin [19] (n = 159 asthmatic patients), but it is smaller than the samples described by Sasaki et al. [17] (n = 192 asthmatic Caucasian patients), Sharma et al. [21] (n = 216 asthmatics) and Deindl [18] (n = 261 asthmatics). Therefore, inconsistent results (at least for 314C/T polymorphism of *HNMT *gene) might have arisen from insufficient sample size and power which is further supported by our demonstration that increasing sample size by combining data from different samples may be useful in validating the existing results by increasing power.

## Conclusions

In conclusion, we report here an association of *HNMT *functional polymorphism with allergic asthma which was further confirmed on a larger group of independent studies. However, interpretation should be careful taking into account the ethnic differences between analyzed populations. Moreover, interactions with other polymorphisms associated with histamine metabolism (eg. histidine decarboxylase gene, histamine receptors genes) may influence the risk of asthma development.

## Competing interests

The authors declare that they have no competing interests.

## Authors' contributions

AS –participated in the study design, recruited the control group, performed genotyping and statistical analysis, participated in interpretation of the results and drafted the manuscript; AB – participated in the study design, recruited patients, collected clinical data; PS – recruited the patients, collected clinical data; AP – recruited the patients, collected clinical data. All authors read and approved the final manuscript.
